# Scraps

**Published:** 1897-12

**Authors:** 


### SCRAPS

PICKED UP BY THE ASSISTANT-EDITOR.

**Clinical Records** (21).—A famous English physician, when quite a young man, was placed in temporary charge of a patient, and, full of the weight of his unaccustomed responsibility, his countenance grew longer and longer. When he was leaving one day, the lady's husband followed him. "I greatly appreciate the anxiety you feel for my poor wife," he whispered, "but please don't let her see it again, for, after you had left the room, she asked me if you were the undertaker."

**Medical Philology** (XXIV.)—Interesting examples of the credulousness of the ancient therapeutists are to be found in connection with the words "Croppe; *cima*" and "Crudde; *coagulum*" (*Catholicon* and *Promptorium*). Mr. Herrtage quotes from Lyte's Dodoens "The decoctions of the toppes and croppes of Dill . . . . causeth wemen to haue plentie of milke" (p. 270). Garden mint "is very good to be applied vnto the breastes that are stretched foorth and swollen and full of milke, for it slaketh and softeneth the same, and keepeth the mylke from quarring and crudding in the brest" (p. 246).

**Medical "Bits of Old Bristol"** (5).—In the *Journal* for September, 1894, I quoted from the Rev. W. Hunt's *Bristol* some of the results of the visit of the plague to this city in the fourteenth century.

There is every reason to believe that the pestilence was brought to the Dorsetshire seaports by sailors returning from the siege of Calais. The Rev. A. Dimock—whose article, "The Great Pestilence: a Neglected Turning Point in English History" (*Gent. Mag.*, Aug., 1897), is well worth reading—thinks that it was easy for travellers over the high roads of Dorsetshire and Somersetshire to have taken it with them to Bristol. But as Bristol was visited perhaps earlier than the West Country inland towns, it is more likely that it was brought direct to the port by sailors. Mr. Dimock says it first appeared in Bristol on August 15th, 1348, thus dating its invasion earlier than Mr. Hunt, who names June, 1349, as the probable time of its first appearance here.

**Nursing Made Easy.**—The Paris correspondent of the *Lancet* writes: "Summoned to attend the child of one of my patients the other day, I found that, alarmed by baby's serious state, a French *confrère* had already been sent for, and left directions which a neighbouring French druggist had translated for the benefit of the English nurse in charge. The document is too amusing to be passed over, and I therefore make no apology for transcribing it, together with the necessary explanation within parentheses supplied by myself. (1) To distend the children of other children. (To isolate the baby from her sister). (2) Not many flower in the eat and not give him that milk prepared. (Suppress farinaceous food and also the milk as hitherto prepared). (3) Before the col of children une éponge warm. (Apply a hot sponge to the child's throat). (4) Every body that have occupation of children wild vhach the hands in liquor of Van Swieten. (Every person coming in contact with the child to wash his hands in Van Swieten's solution). (5) All the linen deteriored shall be whach in solution before londres. (All soiled linen to be washed in the solution before being sent to the laundress)."

**Medical Missions.**—In an address recently delivered at Guy's Hospital, Dr. Symes Thompson said that it is quite impossible to fail to realise how sympathetic and harmonious are the relations which now exist between science and theology. There was a time, not very far distant, when the representatives of the one regarded themselves rather as foes than as friends of the other; but, due in no little measure to the wonderful influence of one great Archbishop who not long ago passed into the Church at Rest, a happier relation now exists. It was not to be wondered at that there was a lack of harmony between the two, when the Church was so dogmatic that it insisted upon positions which it was impossible for thoughtful and intelligent men to hold; nor, on the other hand, was it to be wondered at that the Church should rebel against the shallow dogmatism which was not unusual among the followers of science. We have come to a time when greater breadth and accuracy of knowledge have taken away this arrogance and given us in its place humility, which is not likely to take us into such difficulties. Dr. Thompson alluded to the fact that the University of London has supplied the Universities' Mission with two out of its eight bishops, and then went on to urge the wisdom of encouraging the best of our young medical men to go out into the Mission field for three or four years, and not necessarily to give up their lives to the work. They would then be able to deal successfully with the maladies which fall under their notice, and come back again without their health undermined by the unhealthy conditions to which they had been exposed.—*Central Africa.*

At a recent Chapter of the Guild of St. Luke a discussion was held on the proposed residential College for those intending to go as Medical Missionaries in connection with the Church of England. It was proposed to found a College in London at which students studying medicine at the London hospitals might live, a certain number of whom would be supported by the Guild or other societies, and when qualified would be expected to go as Medical Missionaries, but other students who had no such intention would be received. In view of the proposed reconstruction of the University of London the scheme is of special interest, as it may contain the germ of a class of residential colleges. The College of St. Luke would be governed by a principal, who would be a medical man, and by a chaplain. The Rev. C. H. Rouse, of 49 Bolsover Street, London, W., is the Secretary.—*Church Family Newspaper.*

---

I have received from Dr. Alfred Harvey and from Dr. George F. Rossiter some interesting communications in reference to "Arepo tenet opera rotas," for an explanation of which I asked in the last number. They both point out that the sentence should begin with "Sator," as it is printed in a footnote in the 1813 quarto edition of Brand. The words would then form a "reciprocal" sentence reading the same forwards and backwards. Other instances of such Latin sentences are given in D'Israeli's *Curiosities of Literature* (Routledge's edition, 1866, p. 112). Dr. Harvey says the sentence is "a meaningless jingle of words," and merely forms a charm which can be arranged in a square, thus—

| | | | | |
|---|---|---|---|---|
| S | A | T | O | R |
| A | R | E | P | O |
| T | E | N | E | T |
| O | P | E | R | A |
| R | O | T | A | S |

It will be seen from this that, as Dr. Rossiter mentions, "the first letter of each word makes the first word, the second of each word makes the second word, and so on." He adds that "if the sentence is written twenty-five times in lines one below another it will read not only the same left to right or right to left, but also diagonally, upwards or downwards, left or right."

I have somewhere seen instances of "reciprocal" sentences in English, but have forgotten where. Perhaps some reader will be able to supply some such sentences.

I am still without any explanation of "a.g.l.a.," "bhurnon," and "blictaono."

